# The full-length cell–cell fusogen EFF-1 is monomeric and upright on the membrane

**DOI:** 10.1038/ncomms4912

**Published:** 2014-05-28

**Authors:** Tzviya Zeev-Ben-Mordehai, Daven Vasishtan, C. Alistair Siebert, Kay Grünewald

**Affiliations:** 1 Oxford Particle Imaging Centre, Division of Structural Biology, Wellcome Trust Centre for Human Genetics, University of Oxford, Oxford OX3 7BN, UK

## Abstract

Fusogens are membrane proteins that remodel lipid bilayers to facilitate membrane merging. Although several fusogen ectodomain structures have been solved, structural information on full-length, natively membrane-anchored fusogens is scarce. Here we present the electron cryo microscopy three-dimensional reconstruction of the *Caenorhabditis elegans* epithelial fusion failure 1 (EFF-1) protein natively anchored in cell-derived membrane vesicles. This reveals a membrane protruding, asymmetric, elongated monomer. Flexible fitting of a protomer of the EFF-1 crystal structure, which is homologous to viral class-II fusion proteins, shows that EFF-1 has a hairpin monomeric conformation before fusion. These structural insights, when combined with our observations of membrane-merging intermediates between vesicles, enable us to propose a model for EFF-1 mediated fusion. This process, involving identical proteins on both membranes to be fused, follows a mechanism that shares features of SNARE-mediated fusion while using the structural building blocks of the unilaterally acting class-II viral fusion proteins.

## Abstract (web summary)

Cell–cell fusion in *Caenorhabditis elegans* is mediated by EFF-1 and AFF-1 proteins. Here, the authors present an electron cryomicroscopy 3D reconstruction of EFF-1 in the membrane, and combine snapshots of membrane fusion *in vitro* with a recently reported crystal structure to propose a mechanism for the fusion process.

## Graphical abstract

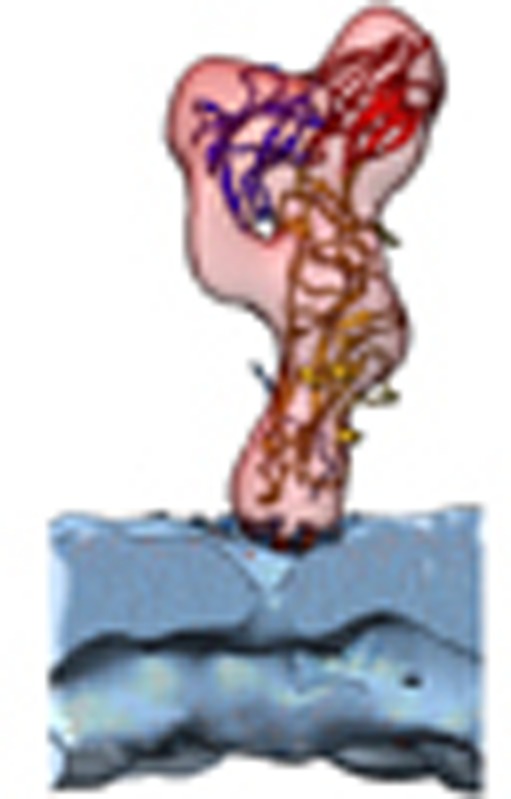

Cell–cell fusion is the principal mechanism by which all multicellular organisms merge cells. It is required in biological processes as diverse as fertilization, development, immune response and tumorigenesis^1^. Biological lipid bilayers do not fuse spontaneously^2^,^3^,^4^. Specific membrane proteins, called fusogens, are required to modulate lipid-bilayers and thereby facilitate membrane merging. The energy required for this remodeling typically comes from protein–lipid interactions, protein re-folding and protein–protein interactions^3^,^5^. A number of specific cell–cell fusogens have been identified^6^ and although some of them have been characterized in detail, structural information has remained elusive.

The first family of *bona fide* extracellular cell–cell fusion proteins was identified in *C. elegans*. The founding members of this cell–cell fusion family (FF) are the epithelial fusion failure 1 (EFF-1) and the anchor-cell fusion failure 1 (AFF-1) proteins^7^,^8^ (Supplementary Fig. 1), both of which are named after their respective knockout phenotypes. Both proteins have been shown to be sufficient to fuse cells *in vivo*^9^, and induce fusion of insect and mammalian cells in cell culture when expressed ectopically^10^. FF-mediated fusion appears to be bilateral and homotypic, that is, dependent on the presence of FF proteins on both fusing membranes^10^. Recently, it was reported that cells expressing AFF-1 and EFF-1 can be infected by pseudotype Rhabdovirus particles when the native viral fusion protein is replaced with the respective fusogen^11^. Furthermore, it has been shown that EFF-1 and AFF-1 are able to functionally complement each other during pseudotype rhabdovirus entry^11^. Although AFF-1 and EFF-1 share some common functional principles, each also has distinct behavioural characteristics. The fusion capacity of each protein differs; AFF-1 appears to be more efficient in mediating fusion than EFF-1, both *in vivo* and in heterologous cells^8^. EFF-1 has also been shown to be crucial in neuronal cell membrane sculpturing^12^.

We developed a novel system that incorporates natively anchored, topologically correct membrane proteins on cell-derived membrane vesicles and used this system to study EFF-1 and AFF-1 by electron cryo microscopy (cryoEM) and cryo tomography (cryoET). The visualization of FF-mediated vesicle fusion confirmed that membrane merging followed the fusion-through-hemifusion pathway and that some of the membrane-merging intermediate states are asymmetric. The three-dimensional (3D) structure of EFF-1 on the membrane was determined by sub-volume averaging. It revealed a membrane protruding, asymmetric, elongated monomer that likely represents the pre-fusion form of EFF-1.

## Results

**Figure 1:**
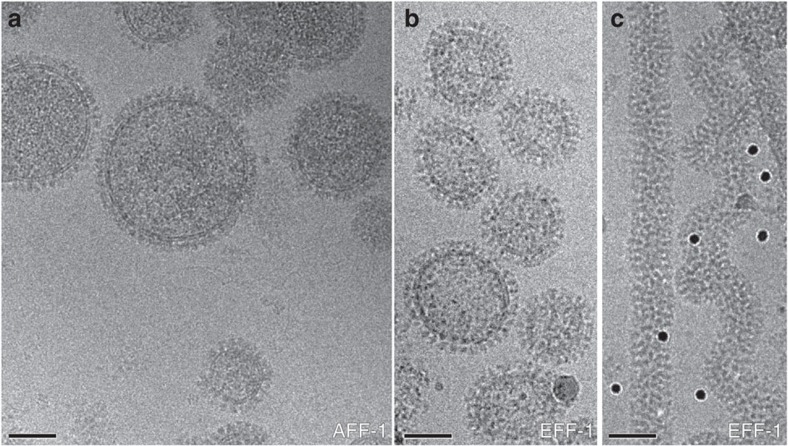
Membranous vesicles with integral epithelial fusion failure 1 (EFF-1) or anchor-cell fusion failure 1 (AFF-1) exhibited different morphologies. (**a**) Electron cryo microscopy (cryoEM) projection image of vesicles with full-length AFF-1. Vesicles harvested 48 h post transfection were mostly spherical. (**b**,**c**) CryoEM projection images of vesicles with full-length EFF-1 showed two distinct morphologies dependent on the harvesting time. EFF-1 vesicles were mostly spherical when harvested 24 h post transfection (**b**) and were predominantly nanotubes (**c**) when harvested 48 h post transfection. The nanotubes appeared to be frequently twisted and were very densely covered with EFF-1 protein. Actin filaments were not observed inside the tubes, in agreement with the exponentially modified protein abundance index analysis (see Methods and Table 1 for details). Black spherical densities in **c** are 10 nm gold fiducial markers. Scale bars, 50 nm.

To characterize the structure and function of EFF-1 and AFF-1 in the context of the membrane, we have developed an experimental system to display full-length proteins on extracellular vesicles. Vesicles with either AFF-1 or EFF-1 protein in the membrane were collected from the culture medium of adherent mammalian cells, at 24 or 48 h post cell transfection with the corresponding gene, and purified by differential centrifugation (see Methods for details). Transfected cells secreted large numbers of vesicles of variable size and highly variable shape (Fig. 1 and Supplementary Fig. 2). These membranous vesicles were uniformly covered with a ~12- to 14 nm-thick protein layer that appeared to consist of discrete densities protruding radially outwards from the membrane, confirming that the correct topology of the proteins was preserved. In control vesicle preparations, where the cells were transfected with an expression plasmid for cytosolic yellow fluorescence protein (YFP), about two orders of magnitude less vesicles were secreted. These vesicles could be seen to display only very small, extra-membranous densities, which were clearly different from those observed on FF vesicles (Supplementary Fig. 2). This indicates that our experimental system is highly suitable for displaying topologically correct membrane proteins, and that vesicle secretion is induced by expression of FF proteins.

The specific enrichment and the identity of the proteins incorporated within the respective vesicles harvested 48 h post transfection (p.t.) were validated by SDS–polyacrylamide gel electrophoresis (Supplementary Fig. 2d) and by mass spectrometry using an exponentially modified protein abundance index (emPAI) analysis^13^,^14^ (Table 1). The emPAI verified that the corresponding FF protein is the most abundant protein in the respective vesicle preparation, which correlated with the SDS–polyacrylamide gel electrophoresis results. No other membrane proteins were detected and most of the other proteins found were contaminants, either from the serum added to the cell culture or from the transfection reagent. Notably, the emPAI clearly indicated that the AFF-1 vesicle preparation contained substantial amounts of actin (~17%), whereas the EFF-1 vesicle preparation contained significantly less (~3% actin; Table 1).

AFF-1 vesicles harvested at either 24 or 48 h p.t. exhibited consistent spherical morphologies. In contrast, EFF-1 vesicles exhibited radically different morphologies dependent on the harvesting time (Fig. 1 and Supplementary Fig. 2). Spherical vesicles comparable to the AFF-1 vesicles were mainly observed when vesicles were collected 24 h post cell transfection. EFF-1 vesicles harvested 48 h p.t. were predominantly composed of nanotubes (Fig. 1c). These nanotubes were several micrometres long (Supplementary Fig. 2g) with a relatively consistent diameter of ~40 nm. The nanotubes appeared to be frequently twisted and were very densely covered with EFF-1 protein (Fig. 1c). Actin filaments were not observed inside the tubes, in agreement with the emPAI analysis. Frequently, the ends of the nanotubes had larger diameters (Supplementary Fig. 2h). Interestingly, extended incubations of purified spherical EFF-1 vesicles (harvested at 24 h p.t.) at 37 °C did not result in their conversion to nanotubes. The nanotubes observed demonstrate that EFF-1 is able to remodel membranes and that this process occurred in the absence of cytoskeletal filaments. This is in agreement with the described function of EFF-1 in neuronal cell membrane sculpturing^12^. The nanotubes observed here seem to be distinct from the recently reported actin-rich membrane protrusions^15^.

**Figure 2:**
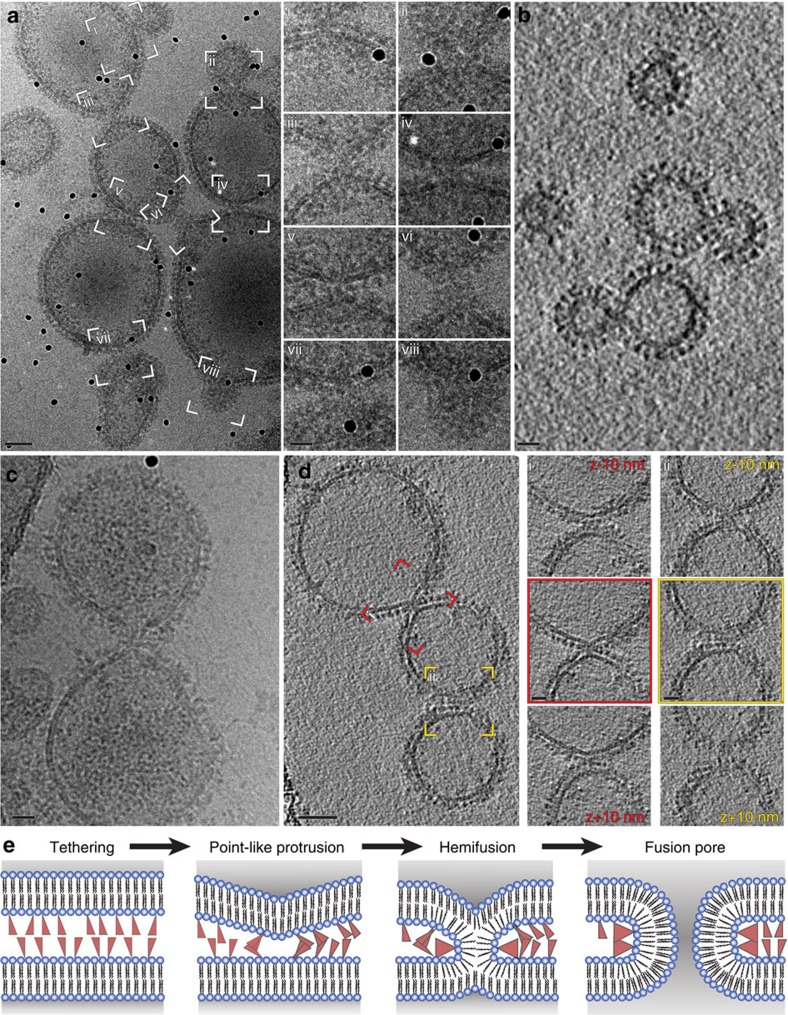
Membrane merging intermediates of epithelial fusion failure 1 (EFF-1) or anchor-cell fusion failure 1 (AFF-1) vesicles. (**a**,**b**) EFF-1 vesicles undergoing fusion. (**a**) Electron cryo microscopy (cryoEM) projection image. Insets on the right show marked fusion sites; rotated and magnified. Different intermediates can be observed, for example, membranes in close proximity (insets marked ii, vii); local high membrane curvature, point-like protrusion (sites iv, v); hemifusion stalk (site iii); putative (elongated) stalk/fusion pore (sites i, vi); late pore expansion state (site viii). (**b**) Slice through a tomogram. (**c**,**d**) AFF-1 vesicles undergoing fusion. (**c**) CryoEM projection image. (**d**) Tomogram slice. Insets on the right show central and tangential slices through fusion sites marked as i and ii; rotated and magnified. Fusion site i (red) shows highly curved membrane on the upper vesicle with density connecting the two membranes. The site is surrounded by protein as can be seen in both the central and the tangential slices (see also Supplementary Fig. 3 and Supplementary Movie 1). Fusion site ii (yellow) shows an early stage of fusion with protein tethering. Black spherical densities are 10 nm gold fiducial markers. Scale bars for **a** and **d**, 50 nm. Scale bars for **b** and **c** and insets, 20 nm. (**e**) Schematic illustration of the fusion-through-hemifusion model for bilayer fusion.

Both EFF-1 and AFF-1 spherical vesicles were fusion active and thus allowed visualization of otherwise highly elusive membrane-merging intermediates. Captured and visualized intermediates included representatives from all previously suggested intermediate states, namely tethered membranes, point-like protrusions, hemifusion stalks and fusion pores^16^ (Fig. 2). Tethered membranes were the most frequently observed intermediate state, with many membranes (*n*=36) found close enough (~20 nm apart) to imply an interaction between their protein layers (~12–14 nm thick on individual vesicles). Membrane fusion sites with high local curvature were relatively rare, but were observed multiple times (*n*=7). Common to all the observed fusion sites was a high abundance of protein (Fig. 2). For the control vesicles with cytosolic YFP, only five were found in close proximity with no high local curvature (Supplementary Fig. 2). The presence of the protein layer made the confident assignment of each observed fusion site to a specific intermediate state more challenging, particularly in projection images. Examining tomographic volumes slice-by-slice provided clearer insight into the fusion states of the membranes (Fig. 2b,d, Supplementary Fig. 3 and Supplementary Movie 1). This analysis revealed that the fusion sites with high local curvature were asymmetric, with one membrane forming a more pronounced point-like protrusion. This is in agreement with results from simulations of membrane fusion mediated by SNARE proteins^17^,^18^ and cryoET of influenza virus fusion with liposomes^19^. Our analyses of different vesicle fusion intermediates confirmed that FF mediated fusion employs the canonical fusion-through-hemifusion mechanism (Fig. 2e) and indicated that some of the membrane-merging intermediate states are asymmetric.

**Figure 3:**
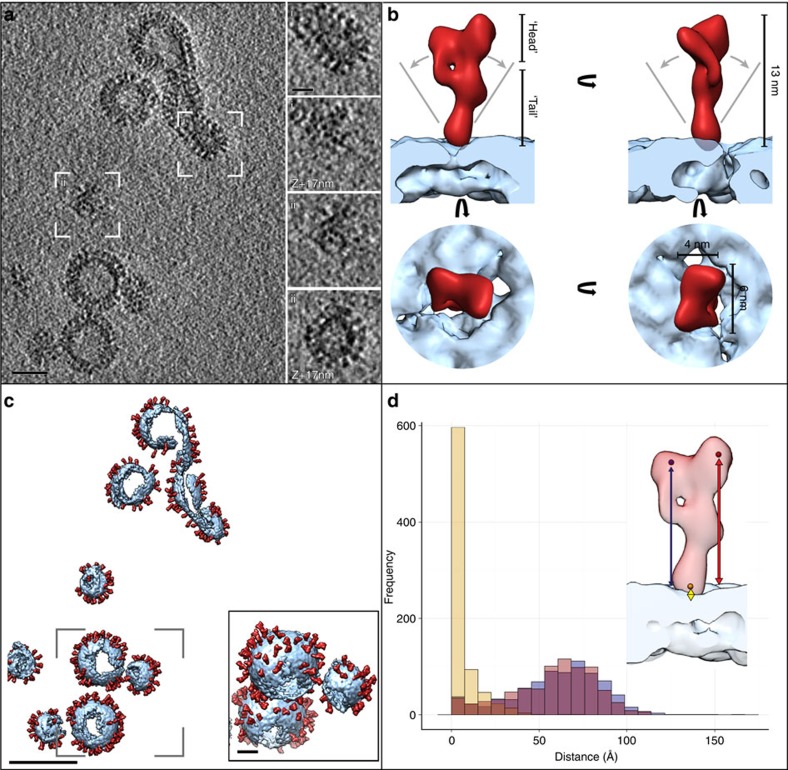
Natively anchored epithelial fusion failure 1 (EFF-1) is an asymmetric elongated monomer. (**a**) Slice through a cryo tomogram of EFF-1 studded vesicles. Insets show magnified central and tangential slices through vesicle fields marked i and ii. Scale bar, 50 nm. Inset scale bar 20 nm. (**b**) Isosurface representation of the sub-volume reconstruction of EFF-1, side and top views are presented. The grey lines indicate the range of observed emerging angles from the membrane (mean 33°, cf. Supplementary Fig. 7). (**c**) Isosurface representation of the tomogram shown in **a**. The EFF-1 sub-volume reconstruction and the membrane were placed in the determined orientation. Monomers appeared to be randomly distributed on the vesicle surface. Inset showing the outlined field magnified and rotated 30°. (**d**) Histogram for all protein particles depicting the distances from three representative points (yellow, red, blue) in the EFF-1 protein to the membrane.

The consistency of EFF-1 proteins on the more spherical vesicles allowed tomogram sub-volumes to be averaged to produce a 3D reconstruction of membrane-anchored EFF-1 (Fig. 3). Sub-volume averaging is a method of aligning and averaging a large number of tomographic sub-volumes that contain the structure of interest, in order to greatly improve the signal-to-noise ratio^20^,^21^. Several hundred sub-volumes were automatically picked at the surface of the vesicles using a local minimum search. These volumes were then computationally extracted, iteratively aligned and averaged in an unbiased, reference-free manner (see Methods and Supplementary Fig. 4). The resulting EFF-1 ectodomain structure (Fig. 3) was determined by Fourier shell correlation (FSC) to have a resolution of 22 and 36 Å at the 0.143 and 0.5 thresholds, based on the consistency of two independent reconstructions from evenly split data sets (gold-standard criterion;^22^ Supplementary Fig. 5). The relative scarcity of EFF-1 fusogens involved directly in the process of vesicle membrane merging (less than 1%; Fig. 2) and their presumed conformational variability precluded the averaging of this protein subset. Our sub-volume averaging workflow was also applied to the EFF-1 on nanotubes resulting in a structure with a broadly similar shape (Supplementary Fig. 6). The limited resolution of this reconstruction was likely due to the fact that the nanotubes were predominantly twisted (Fig. 1c, right example), thus, leading to variability in the local environment of the proteins, for example, between concave and convex membrane areas. Attempts to use the same protocol used for EFF-1 to elucidate the AFF-1 structure did not converge to a consistent average, potentially indicating that AFF-1 may be oriented more tangential to the membrane than EFF-1, or exhibit a larger degree of conformational variability.

Our 3D reconstruction reveals that the natively membrane-anchored ectodomain of EFF-1 has an asymmetric elongated shape (Fig. 3b). It is ~13 nm long and the region most distal to the membrane (‘head’) has a cross-section of ~6 × 4 nm^2^. A narrower region (‘tail’) leads to the membrane. The transmembrane region was only partially resolved, *viz.,* close to the tip of the ectodomain tail. The cytoplasmic domain was not resolved, consistent with it being intrinsically disordered (based on its low-complexity sequence). The mean relative tilt angle of the tail to the normal from the membrane was ~33°, with a standard deviation of ~17°. This tilt was evenly distributed in all radial directions (Supplementary Fig. 7).

To analyse the arrangement of full-length EFF-1 on the membrane, the averaged reconstruction was plotted back into the original tomograms using the determined positions and orientations (Fig. 3c). The distribution of EFF-1 molecules on the vesicle membranes did not appear to conform to any distinct, ordered lattice (Fig. 3c inset). Ectodomains were preferably packed at a minimum distance (centre-to-centre) of ~10 nm from each other.

Recently, the crystal structure of the ectodomain of EFF-1 was determined^23^. This study revealed a trimer with an unexpected similarity to post-fusion class II viral fusion proteins. The protomer of class II viral fusion proteins has an elongated shape with three globular domains that are almost entirely comprised of β-sheets. For the protomer of class II viral fusion proteins, domains I and III are adjacent to each other on one side of the molecule, whereas domain II, with its ‘finger-like‘ shape, forms the other side of the molecule. Both domains II and III are connected to domain I via hinge regions^24^. In viral fusion proteins, domain II contains a hydrophobic fusion loop at the tip of the molecule, but in EFF-1 this loop is highly acidic^23^.

**Figure 4:**
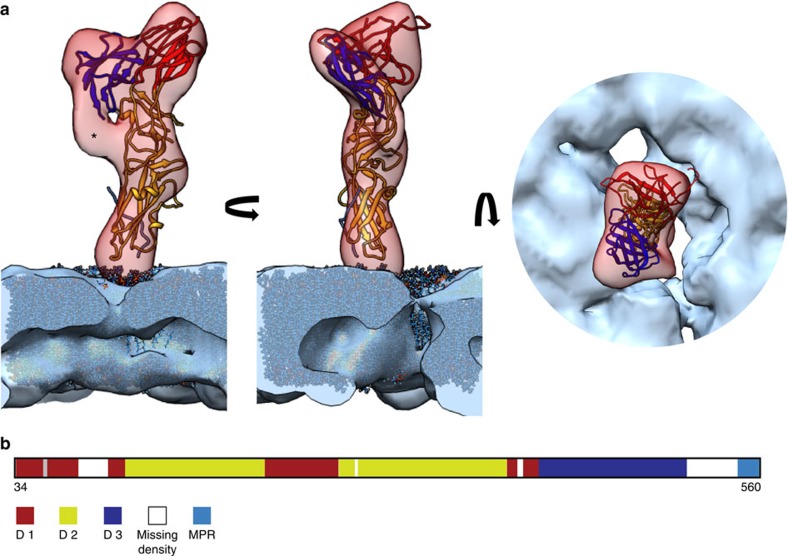
Flexible fitting of the epithelial fusion failure 1 (EFF-1) crystal structure. (**a**) Orthogonal views of the flexible fit of the EFF-1 domains and a lipid bilayer model into the electron cryo microscopic reconstruction. The resulting pseudo-atomic model is shown in ribbon representation and coloured according to the domain boundaries (domain I, red; domain II, yellow, domain III, blue; asterisk, unassigned density). (**b**) Schematic representation of the EFF-1 ectodomain. The domain assignment is based on the crystal structure^23^ (D1, domain I; D2, domain II; D3, domain III; MPR, membrane proximal region). See also Supplementary Movie 2.

**Figure 5:**
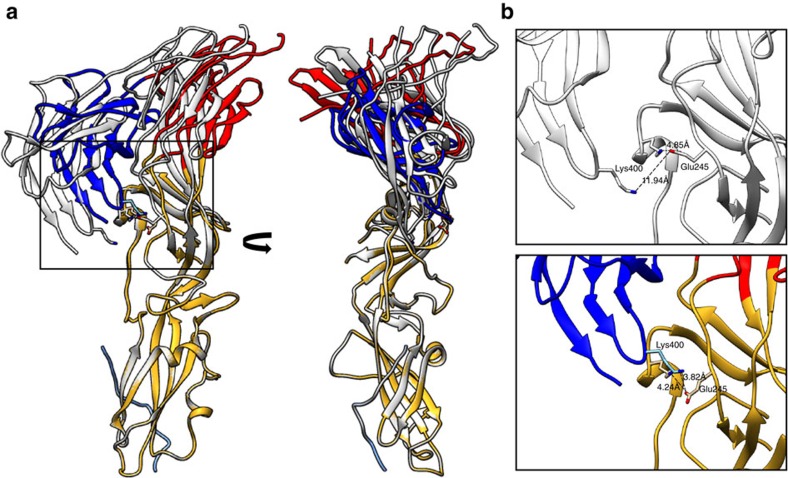
Comparison between the epithelial fusion failure 1 (EFF-1) conformation in the post-fusion and the pre-fusion form. (**a**) Orthogonal views showing the superposition of a protomer from the crystal structure post-fusion trimer (grey) and the pseudo-atomic model derived after flexible fitting into the electron microscopic reconstruction (coloured according to the domains (as in Fig. 4)), using domain II as a reference (see also Supplementary Movie 3). (**b**) Zoom into the area marked in **a** highlighting the formation of a salt bridge between domains II and III.

The ectodomain of EFF-1 observed in the crystal structure is in the post-fusion trimeric conformation, whereas the native membrane EFF-1 structure determined in this study is monomeric. A protomer from the EFF-1 crystal structure was first fitted as one rigid body into the determined EFF-1 EM reconstruction and gave an overall good fit with a cross correlation coefficient of 0.497. To further improve this fit, the protomer was subjected to four iterations of flexible fitting using Flex-EM^25^, with each domain considered a rigid body and flexibility allowed only in the hinge regions. This gave an improved fit with a cross correlation coefficient of 0.502 (Fig. 4, Supplementary Movies 2,3). A lipid bilayer model was fitted into the membrane part of the EM map that was derived by segmentation from step (iii) of the picking and sub-volume averaging pipeline (*cf*. Supplementary Fig. 4). Our structure and the fitting unambiguously revealed that domains I and III are distal to the membrane, and that domain II is proximal to the membrane (Figs 3d and 4a). This orientation with respect to the membrane suggests that the tip of domain II interacts with the lipids of the outer membrane leaflet. The fitting indicates that the interaction is restricted to the lipid head groups (Fig. 4a). It is plausible that this is an electrostatic interaction between the acidic tip of domain II with phosphatidylcholine head groups, thus domain II facilitates the upright anchoring of EFF-1. Intriguingly, using the MOTIF search webservice^26^, the tip of domain II was also found to contain a putative phospholipase A2 aspartic acid-active site motif. Unaccounted density in the EM map between domains II and III (marked by an asterisk in Fig. 4a) most likely accounts for the location of the missing 37 residues from the crystal structure of the stem region leading to the membrane. PCA classification of the sub-volumes using PEET^27^ suggests that the monomer form of EFF-1 constitutes at least the overwhelming majority (Supplementary Fig. 8). We did not find evidence of significant numbers of different stable monomer forms or oligomeric states (Supplementary Fig. 8). The flexible fitting of the domains yielded a ~16° rotation around the domain I–II hinge relative to the crystal structure and a ~19° rotation around the domain I–III hinge (Fig. 5, Supplementary Movie 3). In this conformation, the distance between Glu 245 on domain II and Lys 400 on domain III is substantially shorter than in the EFF-1 crystal structure (reduced from ~12 to ~4 Å), suggesting a possible electrostatic connection. This salt bridge might have a role in stabilizing the pre-fusion conformation and can be a target for testing the validity of our fit in the future.

## Discussion

**Figure 6:**
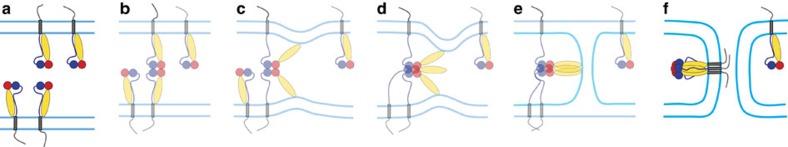
Suggested mechanism for FF-mediated homotypic fusion. (**a**) Pre-fusion monomers anchored approximately perpendicularly to the membrane bilayer (light blue). Domains coloured as in Figs 4 and 5; the transmembrane and cytoplasmic domains are light grey. (**b**) In the tethering step (*cf*. Fig. 2), monomers from opposing cells interact head-to-head in *trans*. (**c**) Formation of a more extended intermediate promotes local membrane curvature. (**d**) A third monomer is then added cooperatively. (**e**) Zippering between the domains I and II of the monomers leads to a fully extended trimeric conformation and to the formation of the hemifusion stalk. (**f**) Relocation of domains III to the trimer outside and the zippering of the transmembrane domains fosters fusion pore opening. To indicate that the fusogen intermediates in steps (**b**–**e**) have not been captured directly, they are shown at a different opacity.

Despite the apparent structural similarity between EFF-1 (and most likely AFF-1) and class II viral fusion proteins^23^, our work demonstrates that EFF-1 seems to promote the merging of the two membranes by a different mechanism that combines aspects of virus–cell fusion and SNARE-mediated fusion. Viral fusion machineries act unilaterally from the viral membrane, whereas FFs act bilaterally, that is, from both membranes and thus in a similar manner to SNAREs^28^. In addition, although class II viral fusion proteins are triggered by acidic pH, the trigger for FF-mediated fusion is still unknown. Our data demonstrate that EFF-1 has a monomeric pre-fusion conformation, protruding upright from the membrane (Figs 1, 2, 3). This is in contrast to the pre-fusion conformation of class II viral fusion proteins, which form dimers that lie flat on viral membranes^24^. In the post-fusion state, all known viral fusion proteins including EFF-1 are trimers^23^,^29^. Combining our results with the bilateral activity of the FF proteins and the mechanism of class II viral fusogen-mediated fusion, we propose a model, depicted in Fig. 6. In the tethering step (Fig. 6b, original data in Fig. 2) monomers from opposing cells interact in *trans*. The interface for this interaction is formed by the contact of the heads, although the exact nature of this interaction remains to be eluded. In the head region there are two missing loops in the crystal structure and thus it is hard to predict the nature of the interface. Interestingly, however, in these missing loops there are two acidic motifs of DDE (amino acid residues 69–71 and 371–373, in domain I) very similar to the one on the tip of domain II (EDD residues 157–159). This acidic motif might have a role in excluding lateral, that is, *cis* interaction of pre-fusion monomers on the same membrane. The head-to-head interaction then triggers a conformational change and the formation of an extended intermediate (Fig. 6c). The acidic EDD motif on the tip of domain II may act to keep domain II anchored to the outer leaflet of the membrane during fusion and thus promote local membrane curvature. A third monomer is then added cooperatively similar to the case in SNARE protein-mediated fusion (Fig. 6d). It is possible that the observed asymmetry in the point-like contacts (Fig. 2) is in part due to the asymmetry of two molecules coming from one membrane and only one from the other. Zippering between the domains I–II of the monomers then likely leads to a fully-extended trimeric conformation (Fig. 6e), similar to pre-hairpin intermediates in viral fusion^30^. Assuming the fusion mechanism follows the viral fusion model, the two membranes are then pulled together concomitant with the protein trimerization, leading to the formation of a hemifusion stalk. The collapse of this extended conformation by the relocation of domains III to the outside of the trimer leads to the EFF-1 post-fusion conformation observed in the crystal structure^23^. This rearrangement, together with the zippering of the transmembrane domains, promotes the opening of the fusion pore (Fig. 6f).

The novel experimental system used here to determine the structure of EFF-1 on the membrane provides a good *in-vitro* platform for further studies to define the trigger of FF-mediated fusion. The natively membrane-anchored EFF-1 structure presented here offers the first glimpse of a cell–cell fusogen in the context of the membrane at molecular resolution. Given the structural homology of EFF-1 to class II viral fusion proteins, one would expect the pre-fusion state of EFF-1 to correspond to the flat dimers typical for this class. However, here we show that the pre-fusion state of EFF-1 is a monomer and projects upright from the membrane. This more exposed conformation may well be crucial to the bilateral action of EFF-1, in contrast to the unilateral action of class II viral fusion proteins. The difference in oligomerization, orientation and mode of action, despite structural similarities, offers intriguing clues into how extracellular, intracellular and viral fusion might have evolved.

## Methods

### Vesicle preparation

Baby Hamster Kidney cells (BHK-21, clone 13, ECACC 85011433) were transfected with either *aff-1* or *eff-1A* gene on pCAGGS plasmid^11^ using lipofectamin (Invitrogen). Following 2 h incubation at 37 °C and 5% CO_2_, the transfection reaction was removed and replaced by Glasgow's minimum essential medium (Invitrogen) supplemented with 2% fetal bovine serum, 2% tryptose phosphate broth and 20 mM HEPES pH 7.4. Transfected cells were allowed to grow for 24 or 48 h at 37 °C and 5% CO_2_. Following the incubation, the medium was collected and cleared from cell debris by centrifugation at 3,000 *g* for 20 min and 4 °C. The vesicles were pelleted through a 20% sucrose cushion at 100,000 *g*, and re-suspended in 25 mM HEPES pH 7.4, 130 mM NaCl. AFF-1 and EFF-1 vesicle preparations were typically done in parallel starting from the same BHK-21 cell culture split for the experiment. For AFF-1 and EFF-1 vesicle preparations harvested 24 h p.t., each 6 independent repeats were performed; for vesicle preparations harvested 48 h p.t., each 19 independent repeats were performed. For every vesicle preparation, two aliquots were analysed by cryoEM.

### Mass spectrometry analysis

For the analysis of protein composition, isolated vesicles with either EFF-1 or AFF-1 were precipitated using chloroform/methanol as described previously^31^ followed by in-solution trypsin digestion^32^. The mass spectrometry analysis was performed by nano ultra-performance liquid chromatography tandem mass spectrometry using a nano Acquity UPLC system coupled to a QTOF premier (Waters) as described previously^33^. Tandem mass spectra were searched against the NCBI database and proteins identified quantified in a relative manner using the emPAI approach as described^13^,^14^; Table 1). For parallel AFF1 and EFF-1 vesicle preparations, mass spectrometry analysis was performed each from two parallel independent preparations and found to be highly similar. The data given in Table 1 are the result from 1 of these experiments.

### CryoEM data collection

Microscopy was performed at either 200 or 300 keV using a TF30 ‘Polara’ electron microscope (FEI) equipped with a QUANTUM 964 post-column energy filter (Gatan) operated in zero-loss imaging mode. A 50-μm C2 aperture and a 20-eV energy-selecting slit were used. Images were recorded on a 4 × 4 k CCD camera at a nominal magnification of 95000 X resulting in a calibrated pixel size of 0.38 nm at the specimen level. Tilt series were collected at 200 kV using SerialEM^34^ at a defocus of −2 μm in three- or four-degree increments covering an angular range from −60° to 60°. The total electron dose for the tilt series was kept between 60 and 80 electrons per Å^2^.

### Tomographic reconstructions

Tomographic reconstructions were calculated in IMOD^35^ using weighted back-projection^36^.

### Sub-volume picking

Sub-volumes were picked using a local minima search on 4x binned and Gaussian-filtered versions of the tomograms. All local minima with intensities lower than two standard deviations below the mean, and within ~150 Å of the manually segmented vesicle membrane, were considered as particles to be averaged, resulting in 1973 sub-volumes. Initial orientations of the sub-volume ‘boxes’ were approximated as normal to the membrane. Using PEET version 1.9 (ref. 37), five iterations of alignment against the initial average of all 1973 sub-volumes were performed on unmasked particles, in order to refine the picking while aligning the membrane as well as the particle. Nearest neighbour distances were calculated based on the average from this step (Supplementary Fig. 4iii). The 801 sub-volumes giving cross-correlation scores above the mean were subsequently used for sub-volume averaging. Using UCSF Chimera^38^, the orientations and positions of these sub-volumes were visualized concurrently with the filtered tomogram maps to validate the results of the picking process. See also (Supplementary Fig. 4)

### Sub-volume averaging

Averaging was performed using PEET version 1.9 (ref. 37). The 801 picked sub-volumes were split into two groups of 400 and 401 particles (based on even and odd particle indices) for the averaging and the final FSC calculation. For each group, the average of all particles in the group was used as the initial template. Six iterations of refinement of the positions and orientations with successively finer sampling increments while including progressively higher spatial frequency information were applied, with the particles masked to remove the membrane and neighbouring particles. The resulting structures from the two independent refinements were aligned and the resolution determined using FSC (Supplementary Fig. 5).

The final structure, created by refining and averaging all 801 sub-volumes, was low-pass filtered using a Gaussian curve with a width matching the FSC curve. The membrane structure from the initial picking and the final particle structure were then plotted back into their relative positions on the original tomogram.

UCSF Chimera^38^ was used for visualization, rigid body fitting of the crystal structure and preparation of the figures. Isosurface representation was thresholded based on the corresponding volume of a monomer molecular weight (87,000 Å^3^).

## Author contributions

T.Z and K.G. designed the experiments; T.Z. performed the experiments; T.Z., D.V., C.A.S., and K.G. processed and analysed the data; and T.Z. and K.G. wrote the manuscript, and all authors commented on it.

The University of Oxford has filed a patent relating to protein enriched extracellular vesicles and their wide applications, T.Z. and K.G. are inventors on the UK patent application 1313249.3.

## Author information

**Accession codes:** The final EFF-1 reconstructions have been deposited in the Electron Microscopy Data Bank (EMDB) at PDBe ( http://www.ebi.ac.uk/pdbe/emdb/) as EMD_2530, 2531, 2532. The Protein Data Bank accession number for the coordinates of the flexible fitting is 4CYL.

**How to cite this article:** Zeev-Ben-Mordehai, T. *et al.* The full-length cell–cell fusogen EFF-1 is monomeric and upright on the membrane. *Nat. Commun.* 5:3912 doi: 10.1038/ncomms4912 (2014).

## Supplementary Material

Supplementary Figures Supplementary Figures 1-8

Supplementary Movie 1 Computational slices through the electron cryo tomogram capturing membrane merging intermediates (Fig. 2d).

Supplementary Movie 2 Isosurface view of the sub-volume averaged EFF-1 structure (as shown in Fig. 3) with a flexibly fitted protomer of the EFF-1 crystal structure.

Supplementary Movie 3 Morph depicting movement of domains between EFF-1 protomer of the crystal structure post-fusion trimer (PDB: 4OJC) used as initial model and the pre-fusion pseudo-atomic model derived after flexible fitting into the EM reconstruction (coloured according to the domains (as in Figs. 4, 5)).

## Figures and Tables

**Table 1:** Major proteins identified by mass spectrometry in EFF-1 and AFF-1 vesicles preparations and their relative abundance.

					**AFF-1**			**EFF-1**		
Protein name*	**Function**†	**Subcellular location**†	**NCBI Accession**	**Mass (Da)**	**Score**‡	**emPAI**§	**Relative abundance (%)**||	**Score**‡	**emPAI**§	**Relative abundance (%)**||
Protein AFF-1 anchor cell fusion failure-1 (*C. elegans*)	Cell–cell fusion	(Plasma) membrane	gi|193204255	68617	974	2.07	33	ND	ND	
Protein EFF-1, isoform a (*C. elegans*)	Cell–cell fusion	(Plasma) membrane	gi|71982882	75494	ND	ND		1004	1.66	16
Histone H2B homologue	DNA binding	Nucleus	gi|156371481	24545	225	1.78	28	392	1.78	17
Histone cluster 1, H2ag-like	DNA binding	Nucleus	gi|291410763	27347	ND	ND		141	1	10
¶Actin family	Cytoskeleton	Cytoplasm	gi|178045	26147	221	1.05	17	185	0.35	3
Hemoglobin fetal subunit beta	Serum	Secreted	gi|62460494	15963	95	0.78	12	234	1.62	15
Serum albumin	Serum	Secreted	gi|1351907	71244	152	0.2	3	282	0.31	3
Galectin-3-binding protein	Promotes integrin-mediated cell adhesion	Secreted	gi|81861611	65270	272	0.41	7	315	0.63	6
60S acidic ribosomal protein P2	Ribosome	Cytoplasm	gi|133062	4692	ND	ND		72	0.79	8
40S ribosomal protein SA-like	Ribosome	Cytoplasm	gi|296190805	32906	ND	ND		182	0.47	4
Guanine nucleotide-binding protein subunit beta-2-like 1	Molecular transducer	Cytoplasm, peripheral membrane protein	gi|5174447	35511	ND	ND		170	0.56	5
Glyceraldehyde-3-phosphate dehydrogenase-like	Enzyme	Cytoplasm	gi|488563203	35942	ND	ND		190	0.55	5
Laminin-binding protein	Carbohydrate binding	Secreted, nucleus, cytoplasm	gi|34234	31888	ND	ND		197	0.49	5
Tubulin beta-3 chain	Cytoskeleton	Cytoplasm	gi|12963615	50842	ND	ND		134	0.37	3

AFF, anchor-cell fusion failure; EFF, epithelial fusion failure; emPAI, exponentially modified protein abundance index; ND, not detected.

^*^All proteins other than AFF-1 and EFF1 are of non-*C. elegans* origin and are originating either from the BHK cells used to produce the EFF-1 and AFF-1 vesicles or contaminations, either from the serum added to the cell culture or transfection reagent.

^†^Based on UniProt annotation.

^‡^Mascot score for confidence of protein identification is defined as the –log value of the probability P that this assignment is made by chance (*21*).

^§^emPAI analysis (*10, 11*) of EFF-1 and AFF-1 vesicles preparations.

^||^Relative abundance with respect to the proteins with the highest emPAI listed in this table.

^¶^Actin family representing gamma-actin, cytoplasmic actin 2, actin—cytoplasmic 1-like.
