# Supplementary material for: The full-length cell–cell fusogen EFF-1 is monomeric and upright on the membrane
Source: Nat Commun. 2014 May 28;5:3912. doi: 10.1038/ncomms4912 (PMC4050280; doi:10.1038/ncomms4912)
Supplement: Supplementary Figures — 1-8 [file ncomms4912-s1.pdf]

SUPPLEMENTARY FIGURES

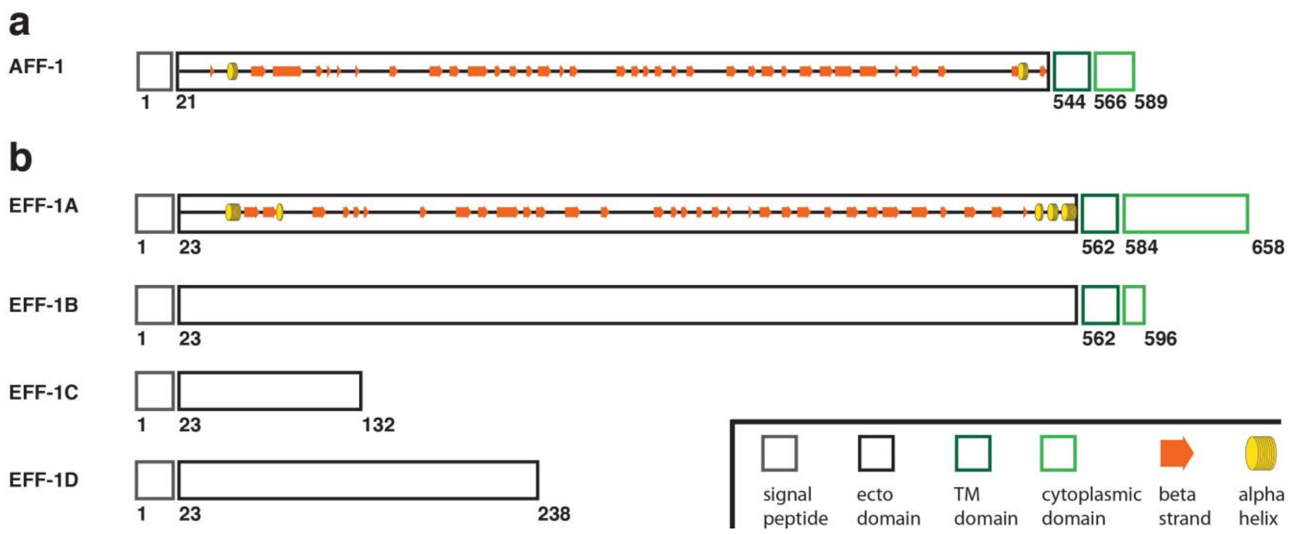

Supplementary Figure 1. Sequence annotation of **a**, AFF-1 and **b,** EFF-1 proteins. Secondary structure predictions for the ectodomains are presented.

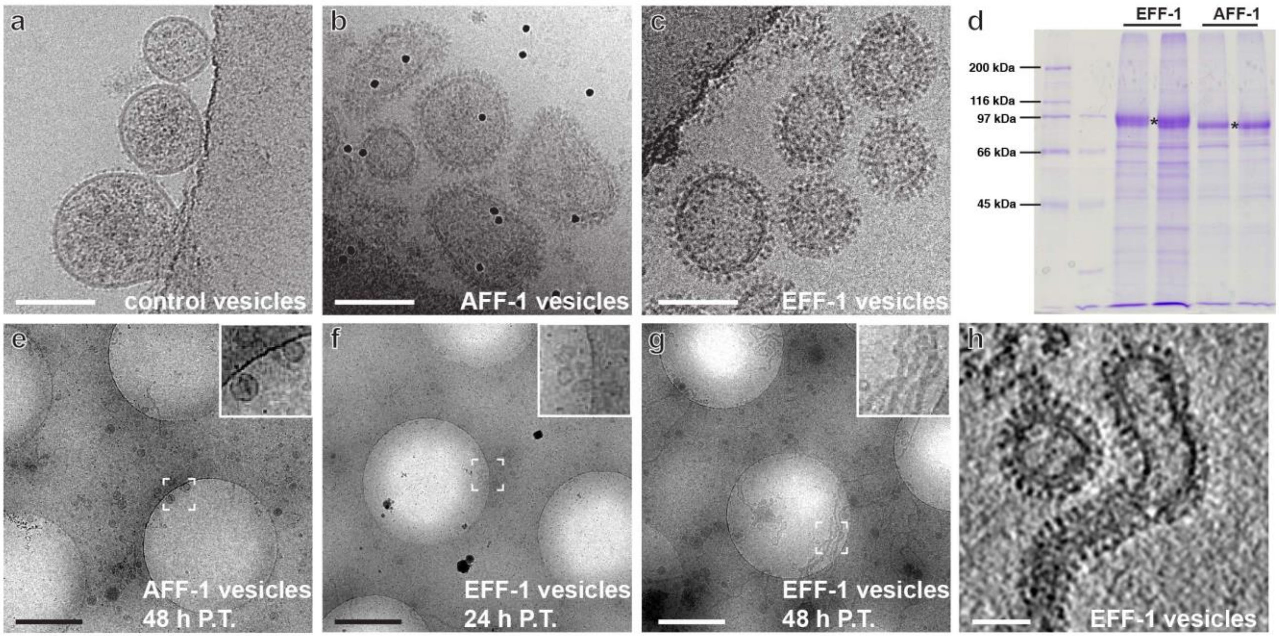

Supplementary Figure 2. Comparison between control vesicles and FF vesicles harvested at different time points post transfection (P.T). **a-c**, CryoEM images of vesicles collected from the culture medium of cells transfected with the expression plasmid for cytosolic YFP (a) full-length AFF-1 (b) and full-length EFF-1 (c). For the YFP control, very small numbers of vesicles were secreted compared to FF-vesicles and the observed densities around the membrane surface were minimal and clearly different from those observed on either AFF-1 or EFF-1 vesicles. **d**, Vesicle preparations of AFF-1 and EFF-1 were loaded on SDS-PAGE. EFF-1 and AFF-1 appear as predominant bands at a molecular weight of ~97 kDa (marked with an asterisk). **e-g**, CryoEM images of vesicles collected from the culture medium at different times P.T. Images were taken at a lower magnification from those in (a-c) to show larger fields of view. AFF-1 vesicles collected 48 h post transfection (e) and EFF-1 vesicles collected 24 h post transfection were mostly spherical. EFF-1 vesicles collected 48 h post transfection were predominantly nanotubes. **h**, Slice through a tomogram of EFF-1 vesicle and the end of a nanotube. Black spherical densities are 10 nm gold fiducial markers. Scale bars for (a-c, h), 50 nm; (e-g), 1 µm.

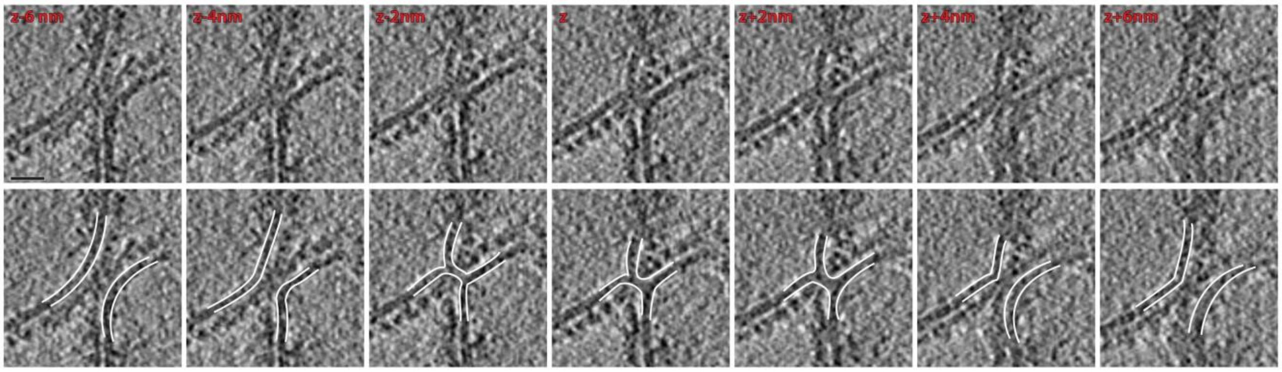

Supplementary Figure 3. Sequential slices from tomogram of the hemifusion site marked (i) in Fig. 2d (see also Supplementary Movie 1). Lower row shows the same slices but overlaid with an outline of the membranes.

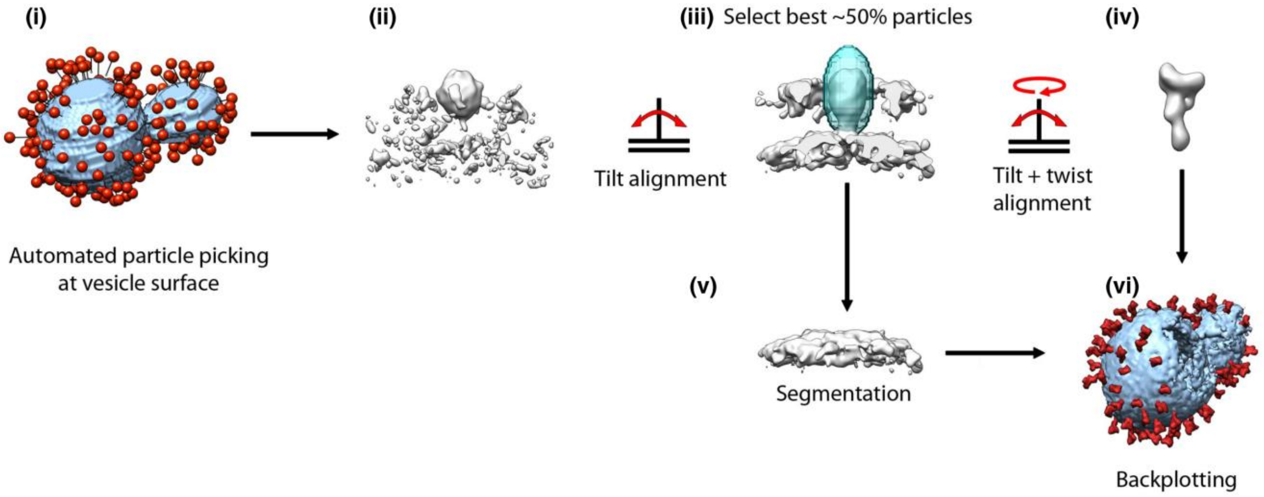

Supplementary Figure 4. Sub-volume averaging workflow. For details see Methods. Panels not to scale.

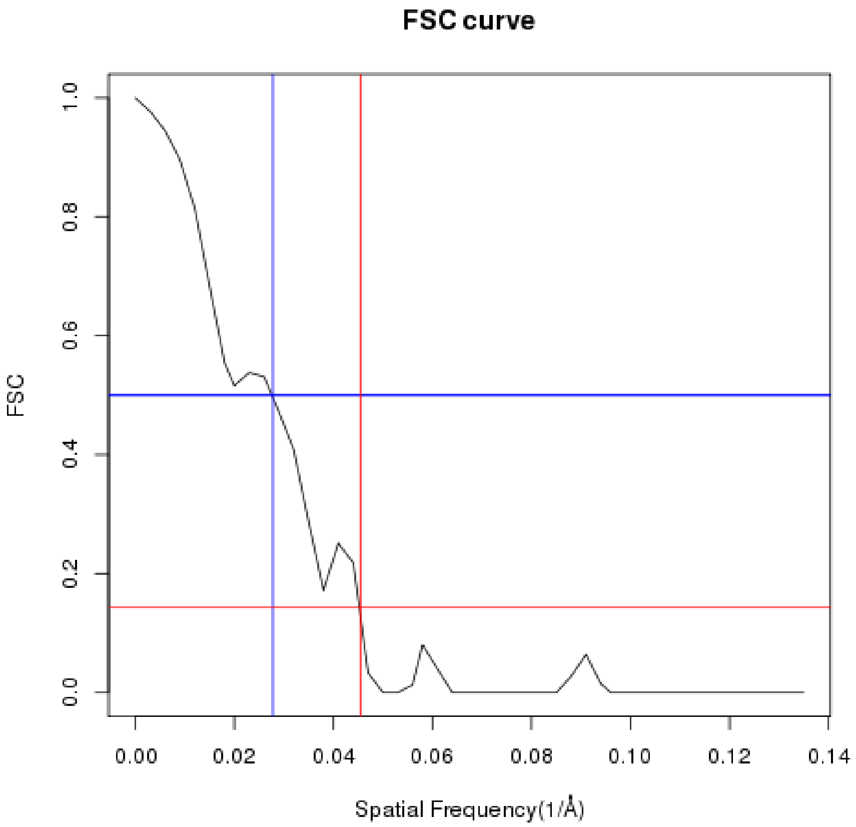

Supplementary Figure 5. Fourier shell correlation (FSC) for two independent reconstructions (gold standard, see Methods for details). The 0.143 and 0.5 cut-offs are marked in red and blue respectively, and indicate resolutions of 22 Å and 36 Å.

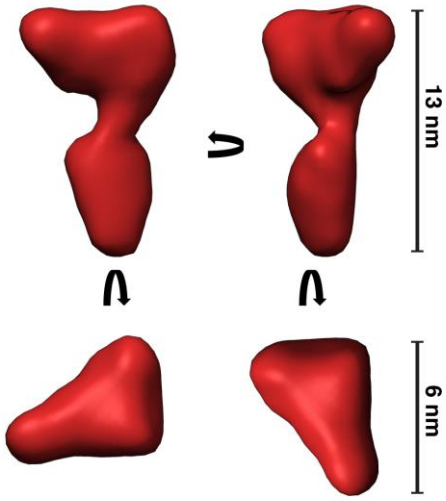

Supplementary Figure 6. Sub-volume average of EFF-1 from nanotubes. Lower resolution (45Å at 0.5 FSC criterion) monomer structure shown in the views as displayed in Fig. 3b (membrane not shown here).

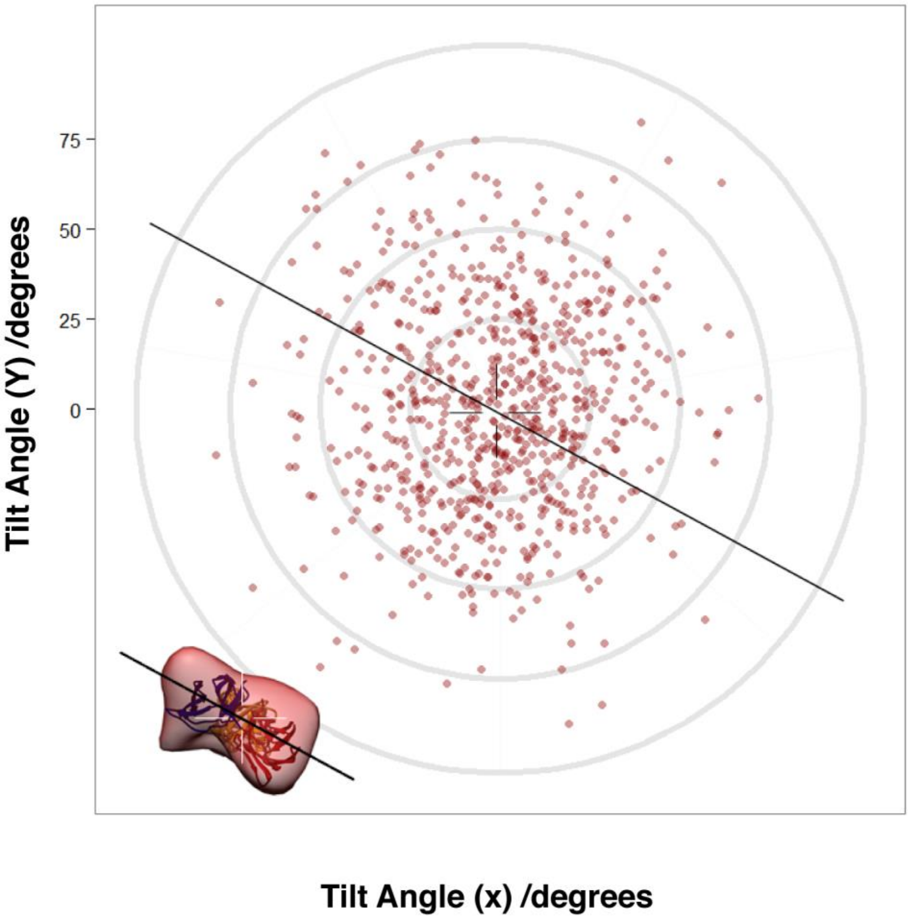

Supplementary Figure 7. Scatter plot displaying the protein tilt angle with respect to the membrane normal. Radial distance from the centre corresponds to the degree of tilt. The direction of tilt is shown in relation to the particle in the bottom left.

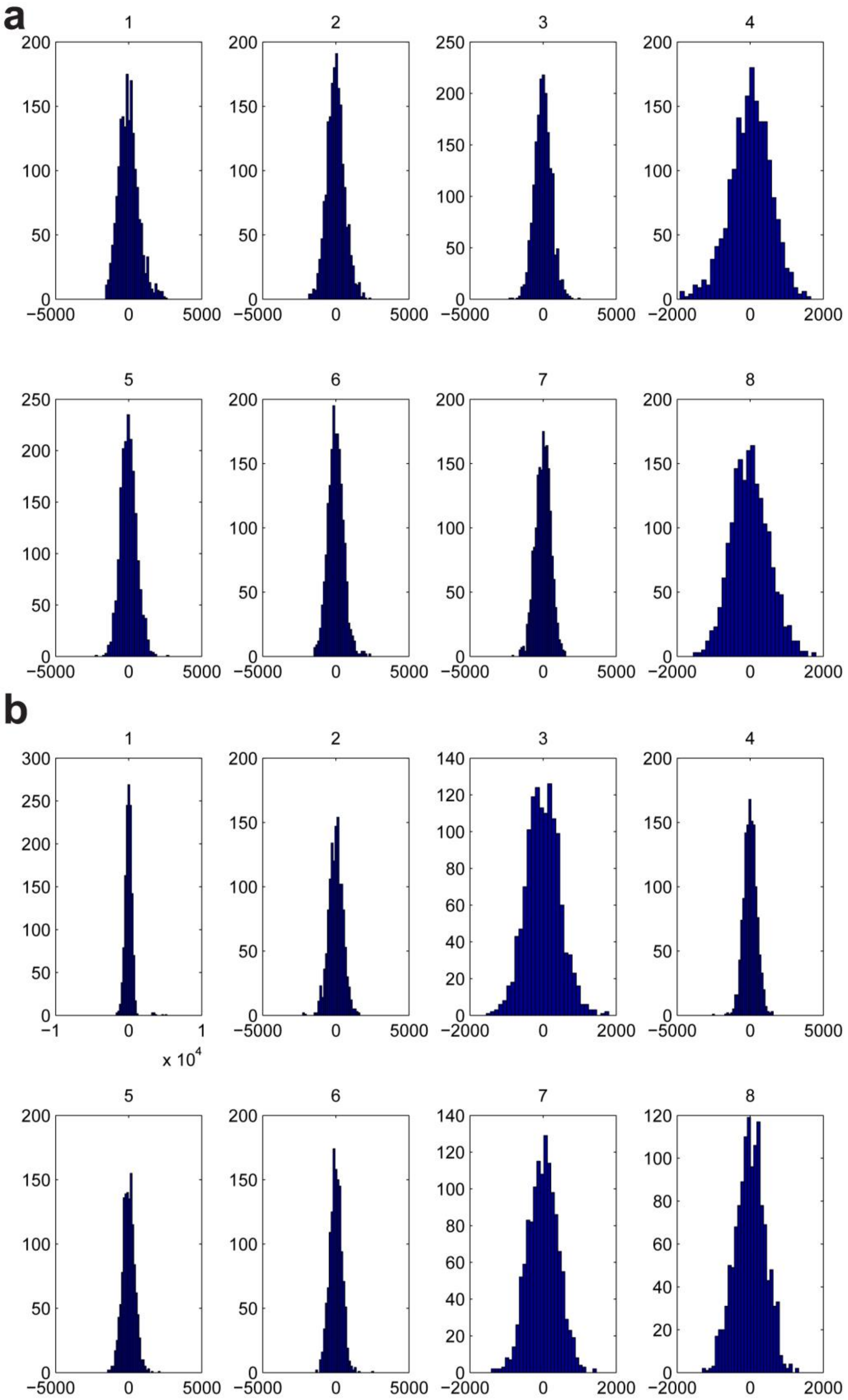

Supplementary Figure 8. Histograms of the particle coefficients along the eight eigenvectors that best describe the variability between the particles. If populations of particles with significantly different conformations existed, separable normal distributions would be visible in one or more of the histograms. **a** Coefficient histograms for EFF-1 particles on spherical vesicles. **b** Coefficient histograms for EFF-1 particles on nanotube vesicles.
